# Analysis of Selected Cardiovascular Biomarkers in Takotsubo Cardiomyopathy and the Most Frequent Cardiomyopathies

**DOI:** 10.3389/fcvm.2021.700169

**Published:** 2021-11-03

**Authors:** Albert Topf, Moritz Mirna, Nina Bacher, Vera Paar, Lukas J. Motloch, Bernhard Ohnewein, Robert Larbig, Janine Grueninger, Uta C. Hoppe, Michael Lichtenauer, Rudin Pistulli

**Affiliations:** ^1^Department of Internal Medicine II, Paracelsus Medical University, Salzburg, Austria; ^2^Devision of Cardiology, Hospital Maria Hilf Moenchengladbach, Moenchengladbach, Germany; ^3^Devision of Cardiology, University Hospital of Muenster, Muenster, Germany

**Keywords:** Takotsubo cardiomyopathy, heart failure, ischemic cardiomyopathy, dilative cardiomyopathy, biomarkers

## Abstract

**Introduction:** Among the causes of *de novo* diagnosed cardiomyopathy, Takotsubo cardiomyopathy (TTC) plays a minor role, with an occurrence of 50,000–100,000 cases per annum in the United States. In clinical practice, a differentiation of a TTC toward an ischemic cardiomyopathy (ICMP) or a dilatative cardiomyopathy (DCMP) appears to be challenging, especially in a subacute setting or in atypical types of TTC.

**Methods:** To investigate this issue, we analyzed serum levels of sST2, GDF-15, suPAR, HFABP, and clinical parameters including echocardiography in 51 patients with TTC, 52 patients with ischemic cardiomyopathy (ICMP) and 65 patients with dilated cardiomyopathy (DCMP).

**Results:** sST-2 seemed to be the most promising biomarker for prediction of a TTC in differential diagnosis to an ICMP (AUC: 0.879, *p* = < 0.001, Cut off values: 12,140.5 pg/ml) or to a DCMP (AUC: 0.881, *p* = < 0.001, cut off value: 14521.9 pg/ml). GDF-15 evidenced a slightly lower AUC for prediction of a TTC in differential diagnosis to an ICMP (AUC: 0.626, *p* = 0.028) and to a DCMP (AUC: 0.653, *p* = 0.007). A differential diagnostic value was found for H-FABP in the prediction of a DCMP compared to TTC patients (AUC: 0.686, *p* = < 0.001). In propensity score matching for left ventricular ejection fraction, sex, and cardiovascular risk factors, differences in the plasma levels of sST2 and H-FABP in the matched cohort of TTC vs. DCMP remained statistically significant. In the matched cohort of TTC vs. ICMP, differences in sST2 also remained statistically significant

**Conclusion:** As medical therapy, long term prognosis, interval of follow-ups, rehabilitation program and recommendations differ completely between TTC and ICMP/DCMP, biomarkers for differential diagnosis, or rather for confirmation of diagnosis, are warranted in cases of cardiomyopathies with unsure origin. sST-2, GDF-15 and H-FABP might facilitate the classification.

## Introduction

Cardiomyopathies are a heterogeneous group of heart muscle diseases that have a major impact on the quality of life, life expectancy und health care costs. Among cardiomyopathies, ischemic cardiomyopathy (ICMP) and dilated cardiomyopathy (DCMP) are the most relevant. Takotsubo cardiomyopathy (TTC) is considered as a primary but acquired cardiomyopathy (1).

Takotsubo syndrome is estimated to appear with about 50,000–100,000 cases per annum in the USA and with similar numbers in Europe (2, 3). In comparison ischemic cardiomyopathy affects over 15.5 million patients in the USA and patients with a DCMP are concerned with a prevalence of 36/100,000 in the USA (4, 5).

DCMP is a primary heart muscle disease characterized by progressive left or biventricular dilation and systolic dysfunction in the absence of hypertension, a significant coronary artery disease and severe valvular disease. Accepted etiological causes are genetic disorders, infections, systemic immune-mediated diseases, toxic, drug-associated, endocrine, metabolic, and peripartal disorders. DCMP represents one of the main reasons for progressive deterioration of biventricular function resulting in a listing for heart transplantation and patients are jeopardized for SCD (6–9).

ICMP is considered as a left ventricular dysfunction in the presence of severe coronary artery disease, including at least either a prior revascularization, an acute coronary syndrome (ACS), a stenosis with more than 75% in the left main stem/the left anterior descending artery or two coronary vessels with more than 75% of luminal stenosis (10, 11). ICMP represents the most common cause of heart failure in the developed world. Despite innovations in patient care, including new antithrombotic drugs and improvements in percutaneous coronary intervention (PCI), the morbidity, and mortality remains high (12, 13).

Takotsubo cardiomyopathy (TTC) is an acute heart failure condition characterized by acute left ventricular deterioration with symptoms indistinguishable from an ACS, but in the absence of a significant coronary stenosis (14). Despite, an incidence of even 7.5% in the female population, 3% of all suspected acute coronary syndromes (ACS) are caused by TTC (15). Emotional and physical stress factors are often reported as triggers and TTC comprises reversible wall motion abnormalities involving apical, midventricular or basal segments of the left ventricle (16). The pathophysiological mechanism of TTC has not been completely understood. There is suspicion that in TTC, the myocardium responds to excessive catecholamine release with myocardial stunning (17).

The majority of TTC patients has a good prognosis, and full recovery with resolution of wall motion abnormalities within 1 month in reported in 96% of TTC patients (18). Nevertheless, the acute phase can be life-threatening (1-2% mortality). There is a 20% risk of congestive heart failure, life-threatening ventricular arrhythmias occur in 8.6% and even left ventricular wall rupture, thrombosis, and cardiogenic shock have been reported (19).

Biomarker determination is implemented in clinical practice with high recommendation in ICMP as well as in DCMP (20). So far biomarker measurements have been focusing in TTC on differential diagnosis toward an acute coronary syndrome (21). In clinical practice, this question remains the predominant issue. Nevertheless, in daily routine, a differentiation of a TTC from an ICMP or a DCMP appears to be challenging, especially in a subacute setting or in atypical types of TTC (22, 23). As medical therapy, long term prognosis, follow-up, rehabilitation program, and recommendations differ completely, biomarkers for differential diagnosis, or rather for confirmation of diagnosis, are warranted.

To best of our knowledge, biomarkers have not been investigated in TTC for a differential diagnosis toward an ICMP and a DCMP.

In this study, we investigated a selected spectrum of novel cardiovascular biomarkers for their differential diagnostic value in TTC. We chose markers already well studied in other cardiovascular diseases (23, 24).

One of the best studied markers with frequent use in clinical practice is soluble suppression of tumorigenicity (sST-2). sST-2 is a member of the interleukin-1 (IL-1) receptor family, which is known to act as a membrane bound receptor (ST2L) but also as a secreted protein (soluble ST-2; sST-2) (25). The functional ligand for the ST2L receptor is Interleukin-33 (IL-33). Local tissue inflammation and necrotic cell death as a danger signal trigger the IL-33 secretion (26). Expressed by cardiomyocytes and cardiac fibroblasts, an excess of sST-2 leads to binding and subsequent reduced bioavailability of circulating cardioprotective ligand IL-33, which reduces apoptosis and improves myocardial function. Furthermore, sST-2 has been identified as a marker of cardiac mechanical strain (27).

Growth-differentiation factor-15 (GDF-15) is a member of the transforming growth factor β-family and has also been postulated as a stress-responsive biomarker of cardiac and vascular disease. GDF-15 expression is up-regulated in the presence of oxidative stress and inflammation (28).

Soluble urokinase plasminogen activator receptor (suPAR) is a proinflammatory marker, which is associated with systemic inflammatory response syndrome, malignancies, and cardiovascular disease. Furthermore, suPAR is expressed in a variety of cells with a role in all stages of atherogenesis—from the initiation of fatty streaks to progression of atherosclerosis and plaque destabilization. Plasma levels of suPAR are associated with atherosclerosis and with individual's risk for cardiovascular disease, type 2 diabetes mellitus, cancer, as well as mortality (29).

Heart-type fatty acid binding protein (H-FABP) is a low molecular weight protein which is expressed in cardiomyocytes. Similar to troponin, H-FABP is released in the presence of myocardial damage, such as ischemia, why it is considered as an early indicator for ischemic heart damage. Increased H-FABP levels at hospital admission are predictive for lethal outcome, as well as for non-fatal cardiac adverse events, even in absence of troponin elevations (30).

The aim of this study is to investigate the differential diagnostic value of novel biomarkers to distinguish TTC from ICMP and DCMP.

## Methods

### Patients and Controls

In this prospective study, we recruited 168 patients with cardiomyopathies. 51 patients with TTC were enrolled, if they fulfilled the Mayo Clinic Diagnostic Criteria for TTC (31). 65 patients with a DCMP and 52 ICMP patients, who were all clinically compensated, were implemented within a routine follow up. ICMP and DCMP were diagnosed and treated in accordance with the European Society of Cardiology criteria (32).

Serum samples of TTC patients were collected within 24 h after the onset of symptoms. Data on clinical presentation, precipitating factors, cardiovascular risk factors, medications and demographics were obtained as well.

### Blood Samples

Blood samples were collected from a cubital vein using a sterile technique under controlled venous stasis. The collection tubes were centrifuged within 20 min after blood collection and the obtained plasma samples were frozen at −80°C until further analysis was performed. Routine blood analysis, according to our clinical standards, was also performed at the time of initial study sample collection.

### Transthoracic Echocardiography

Transthoracic echocardiography at baseline (Philips iE 33 ultrasound system) was used to assess left ventricular ejection fraction (LVEF). Standard echocardiographic views, including parasternal long axis view, parasternal short axis view and apical four chamber view, were acquired as previously published (33).

### Biomarker Analysis

Serum biomarker analysis was performed at baseline. Levels of sST-2, GDF-15, suPAR, and hFABP were measured by using commercially available enzyme linked immunosorbent assay (ELISA) kits (DuoSet ELISA, DY523B, R&D Systems, Minneapolis, MN, USA). ELISA assays were performed in accordance with instructions supplied by the manufacturer. In short, serum samples and standard proteins were added to the multiwell plate coated with the respective capture antibody and incubated for 2 h. Plates were then washed using washing buffer (Tween 20, Sigma Aldrich, USA, and phosphate buffered saline solution). In the next step, a biotin-labeled antibody was added to each well and incubated for an additional 2 h. After incubation, the ELISA plates were washed and a streptavidin-horseradish-peroxidase solution was added. After adding tetramethylbenzidine (TMB; Sigma Aldrich, USA), a color reaction was achieved. Optical density was measured at 450 nm on an ELISA platereader (iMark Microplate Absorbance Reader, Bio-Rad Laboratories, Austria).

### Statistical Analysis

Statistical analysis was performed using GraphPad-Prism software SPSS (22.0, SPSS Inc., USA) and R (version 4.0.2., R Core Team (2013), R Foundation for Statistical Computing, Vienna, Austria; http://www.R-project.org/) with the packages “ggplot2,” “glmnet,” “pastecs,” “Hmisc,” “ggm,” “QuantPsyc,” “Matching,” “MatchIt,” “optmatch,” “RItools” and “Rcpp.” The Kolmogorov-Smirnov test was used to assess distribution of data in the study population. As most parameters and biomarker concentrations were not normally distributed, all values were given as median and interquartile range (IQR). Median values between groups were compared by Mann-Whitney-U test or Kruskal Wallis test with Dunn's *post hoc* test. Correlation analysis was performed using Spearman's rank-correlation coefficient. ROC analysis was performed and an optimal cut-off was calculated by means of the Youden Index. Areas under the curve (AUC) were compared as described by Hanley and McNeil. Propensity score matching was conducted using near neighbor with caliper matching with ε <0.1 σp. A *p* < 0.05 was considered to be statistically significant.

## Results

### Baseline Characteristics

Baseline characteristics of TTC patients and those suffering from ICMP or DCMP are shown in Table 1. TTC patients were significantly older than patients with ICMP (*p* = 0.019) and DCMP (*p* < 0.001). According to the etiology of TTC, female gender was predominant in the TTC subgroup (94.1%). Left ventricular ejection fraction of patients with TTC was insignificantly increased when compared to patients with ICMP (*p* = 0.055), but was significantly higher compared to patients with DCMP (*p* = 0.003). Creatinine plasma levels were significantly increased in patients with ICMP and DCMP compared to TTC patients (*p* = < 0.001).

**Table 1:** Baseline characteristics patients suffering from TTC or ICMP and DCMP, given as median and IQR.

	**TTC**		**ICMP**		***P* = **	**DCMP**		***P =***
	**Median**	**IQR**	**Median**	**IQR**		**Median**	**IQR**	
Age (years)	74.0	62.0–78.0	66.0	56.5–72.8	0.019	55.0	48.3–63.0	<0.001
BMI (kg/m^2^)	24.7	21.8–29.2	27.6	24.2–31.3	0.008	28.5	25.1–33.0	0.001
EF (%)	40.0	35.0–46.0	37.5	29.3–47.0	0.055	35.0	28.0–44.0	0.003
Creatinine (μmol/l)	64.2	59.8–79.2	99.0	81.3–131.0	<0.001	89.0	78.0–122.0	<0.001
LDL (mg/dl)	90.0	75.0–122.0	70.3	61.8–86.1	0.048	95.7	79.5–144.0	0.019
CRP (mg/l)	0.4	0.2–0.9	2.8	2.0–7.5	0.004	3.2	0.0–7.9	0.031
Pro-BNP (pg/ml)	2,866.0	664.6–4,919.8	2,655.0	1,073.0–7,423.0	0.178	3,020.0	475.0–12,855.0	0.389
sST-2 (pg/ml)	24,354.9	13,071.5–47,468.3	8,522.0	6,034.5–10,811.3	<0.001	8,140.0	5,581.5–11,345.7	<0.001
H-FABP (ng/ml)	1.1	0.6–2.3	1.7	0.0–3.8	0.703	2.2	1.3–3.1	0.001
suPAR (pg/ml)	3,076.8	2,350.3–4,118.0	3,681.1	2,534.7–5,072.4	0.063	3,377.4	2,349.4–4,823.6	0.246
GDF-15 (pg/ml)	924.8	610.7–1,529.3	688.8	446.0–987.0	0.028	627.8.8	412.0–947.8	0.007
Smoking	15/51 (29.4%)		21/52 (40.4%)			27/65 (41.5%)		
Hypertension	38/51 (74.5%)		40/52 (76.9%)			24/65 (36.9%)		
Diabetes			21/52(40.4%)			21/65(32.3%)		
Sex (female)	48/51 (94.1%)		8/52 (15.4%)			20/65 (30.8%)		

*p = significance between TTC and ICMP patients or significance between TTC and DCMP*.

Regarding comorbidities, hypertension and diabetes was most frequently represented in ICMP patients, whereas smokers were with highest prevalence in the DCMP group.

### Biomarkers

**Figure 1:**
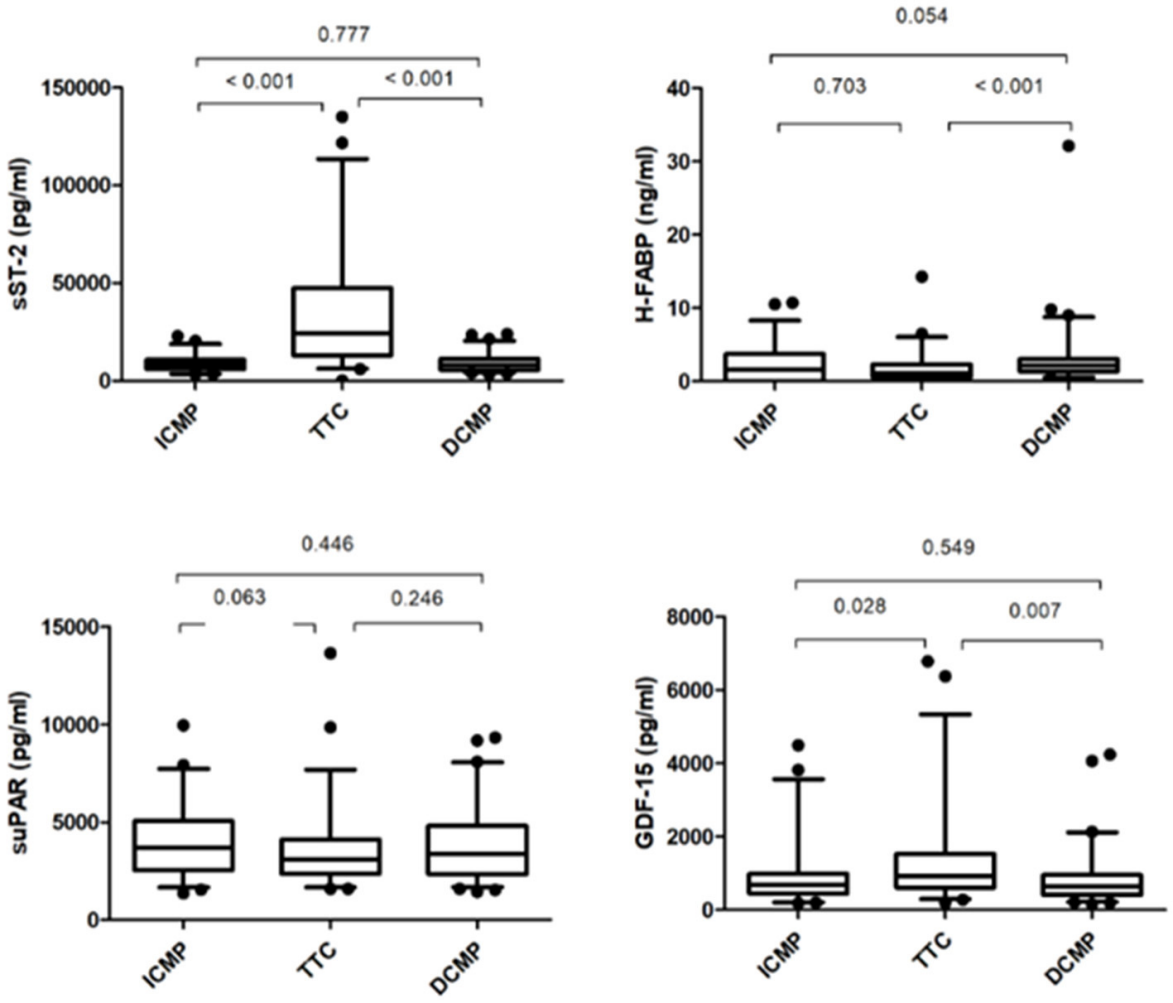
Comparison of biomarker levels between ICMP group, TTC, and DCMP patients.

There was no significant difference among the baseline plasma levels of sST-2, H-FABP, suPAR, and GDF-15 between ICMP and DCMP patients. sST2 was significantly increased in patients with TTC at baseline compared to patients with ICMP and DCMP (*p* = < 0.001, see Figure 1). Whereas the plasma levels of h-FABP between the TTC and the ICMP group did not differ significantly (*p* = 0.703), there was a considerable difference of H-FABP concentrations of TTC patients compared to the DCMP group (*p* = < 0.001). The plasma levels of suPAR did not significantly differ among the subgroups.

The plasma levels of GDF-15 were significantly increased in patients with TTC compared to patients with ICMP (*p* = 0.028) and DCMP (*p* = 0.007). When considering Pro-BNP levels, there was no significant difference among the three subgroups (ICMP vs. DCMP; *p* = 0.680).

### Correlation

Correlations between biomarkers and patient characteristics are depicted in Table 2. Except for suPAR and H-FABP, a correlation of biomarkers with patient age was found. suPAR and H-FABP correlated inversely with left ventricular ejection fraction. Only sST-2 had a weak inverse correlation with BMI. Except for sST-2, all biomarkers correlated with plasma creatinine levels. No correlation of biomarkers with plasma levels of C-reactive protein (CRP) or LDL-cholesterol were found. A strong correlation was found between sST-2, suPAR, GDF-15 and H-FABP.

**Table 2:** Bivariate correlation and point-biserial correlation analysis of baseline characteristics and biomarkers.

	**sST-2**		**suPAR**		**GDF-15**		**H-FABP**	
	***rs***	***p =***	***rs***	***p =***	***rs***	***p =***	***rs***	***p =***
Age (y)	0.332	<0.001	0.089	0.265	0.407	<0.001	0.123	0.122
BMI (kg/m^∧2^)	−0.194	0.015	0.039	0.627	−0.082	0.307	0.145	0.071
EF (%) Creatinine (μmol/l)	−0.051 −0.124	0.516 0.138	−0.164 0.260	0.037 0.002	−0.070 0.259	0.377 0.002	−0.202 0.364	0.010 <0.001
CRP (mg/dl)	−0.061	0.507	0.100	0.271	0.174	0.055	0.084	0.354
LDL (mg/dl)	0.070	0.502	−0.154	0.136	−0.080	0.443	0.192	0.063
sST−2 (pg/ml)	1.000	0.000	0.227	0.003	0.588	<0.001	0.168	0.030
GDF-15 (pg/ml)	0.588	<0.001	0.463	<0.001	1.000	0.000	0.455	<0.001
H-FABP (ng/ml) suPAR (pg/ml)	0.168 0.227	0.030 0.003	0.410 1.000	<0.001 0.000	0.455 0.463	<0.001 <0.001	1.000 0.410	0.000 <0.001

### ROC Analysis

**Figure 2:**
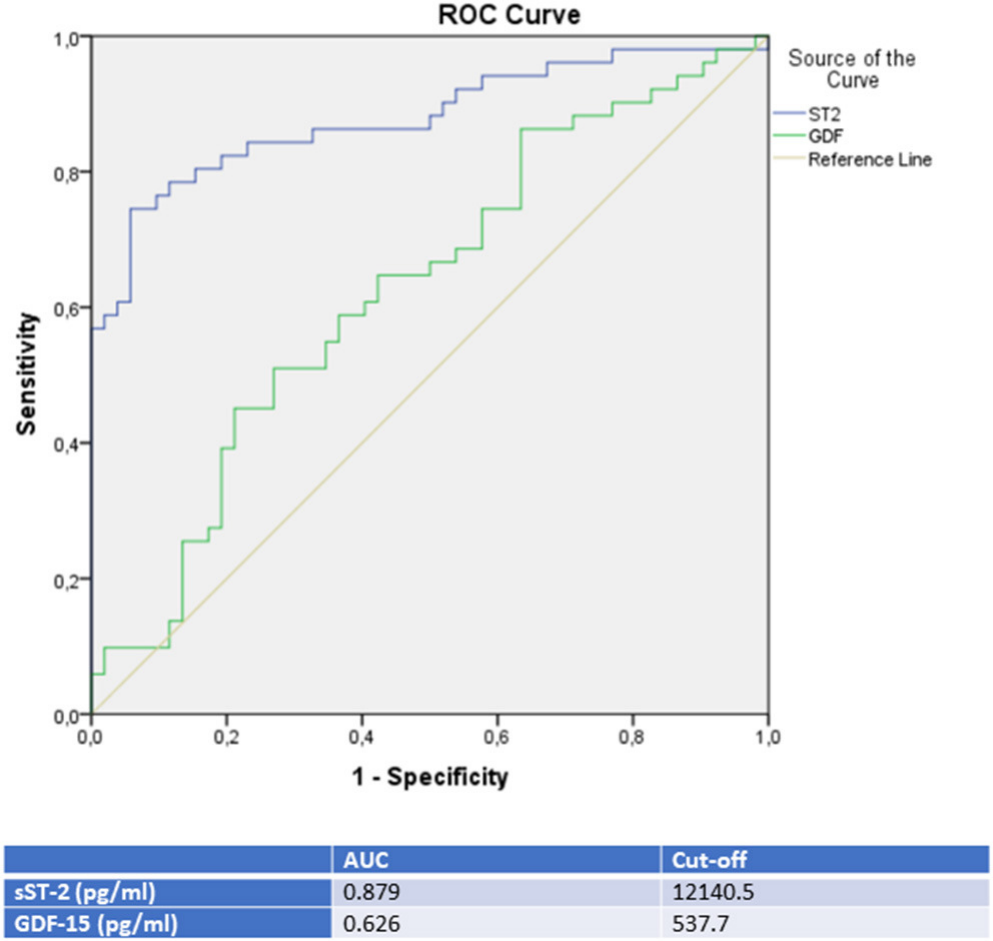
ROC-Curves and cut off scores for sST-2 (ST2) and GDF-15 (GDF) prediction of TTC in the total cohort (including patients with TTC and ICMP).

**Figure 3:**
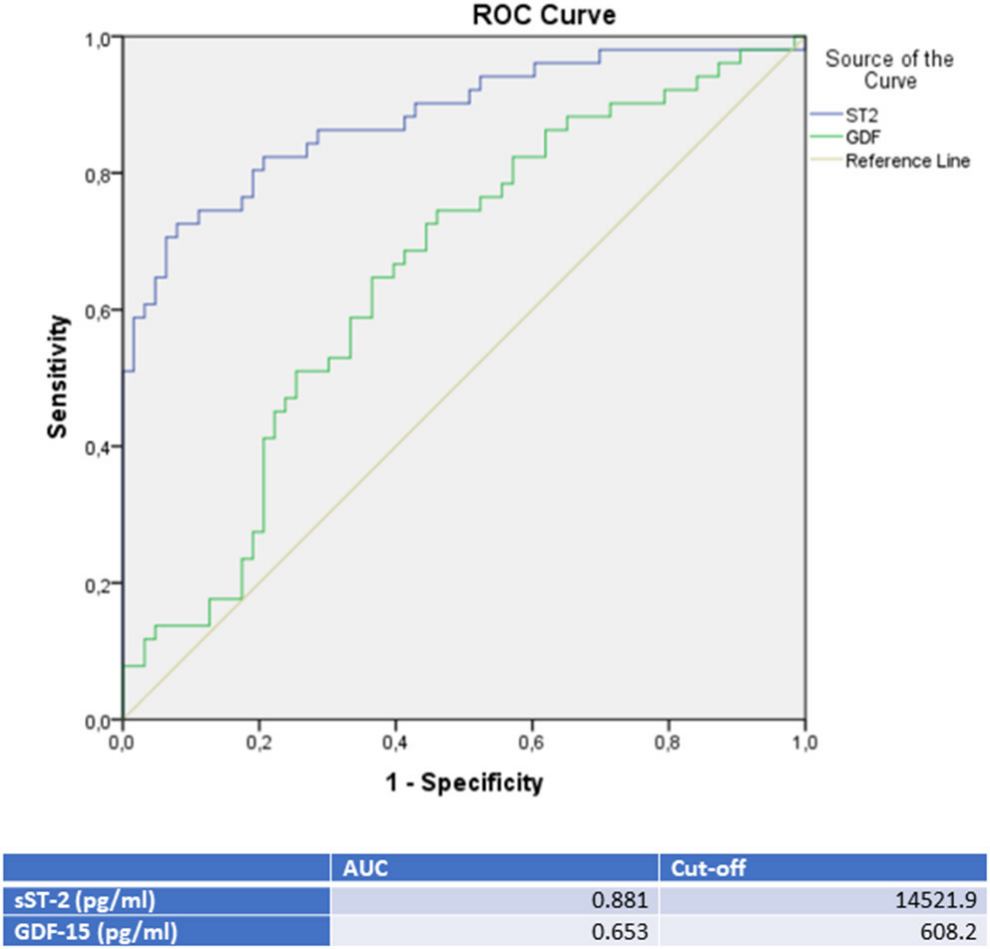
ROC-Curves and cut off scores for sST-2 (ST2) and GDF-15 (GDF) prediction of TTC in the total cohort (including patients with TTC and DCMP).

Moreover, a ROC analysis was performed and AUC was calculated for GDF-15 and sST-2 levels as differential diagnostic indicators for patients presenting with *de novo* heart failure in the case of either TTC, ICMP or DCMP. In this analysis, sST-2 and GDF-15 were identified as the paramount biomarkers for identification of a TTC in differential diagnosis to either an ICMP (see Figure 2 and Table 3), to a DCMP (see Figure 3) or to both cardiomyopathies (see **Figure 5**).

**Table 3:** Rates for sensitivity, specificity, positive and negative predictive value for all tested biomarkers in TTC and ICMP patients.

**TTC**	**Sensitivity**	**Specificity**	**PPV**	**NPV**
GDF-15	86.3%	36.5%	57.1%	73.1%
sST-2	78.4%	84.6%	83.3%	80.0%

sST-2 seemed to be the most promising biomarker for prediction of a TTC in differential diagnosis to an ICMP (AUC: 0.879, *p* = < 0.001) or to a DCMP (AUC: 0.881, *p* = < 0.001). An optimal cut off for diagnosis of TTC by means of the Youden—Index was calculated as 12,140.5 pg/ml (sensitivity: 78.4%, specifity: 84.6%, PPV: 83.3%, NPV 80.0%) for identification of a TTC in comparison to ICMP and 14521.9 pg/ml (sensitivity: 74.5%, specifity: 88.9%, PPV: 82.6%, NPV: 81.4%) for differential diagnosis to a DCMP.

Compared to sST-2, GDF-15 evidenced a slightly lower AUC for prediction of a TTC in differential diagnosis to an ICMP (AUC: 0.626, *p* = 0.028) and to a DCMP (AUC: 0.653, *p* = 0.007). An optimal cut off for diagnosis of TTC by means of the Youden—Index was calculated as 537.7 pg/ml (sensitivity: 86.4%, specifity: 36.5%) for identification of a TTC in comparison to ICMP and 608.2 pg/ml (sensitivity: 76.5%, specifity: 47.6%) for differential diagnosis to a DCMP.

**Figure 4:**
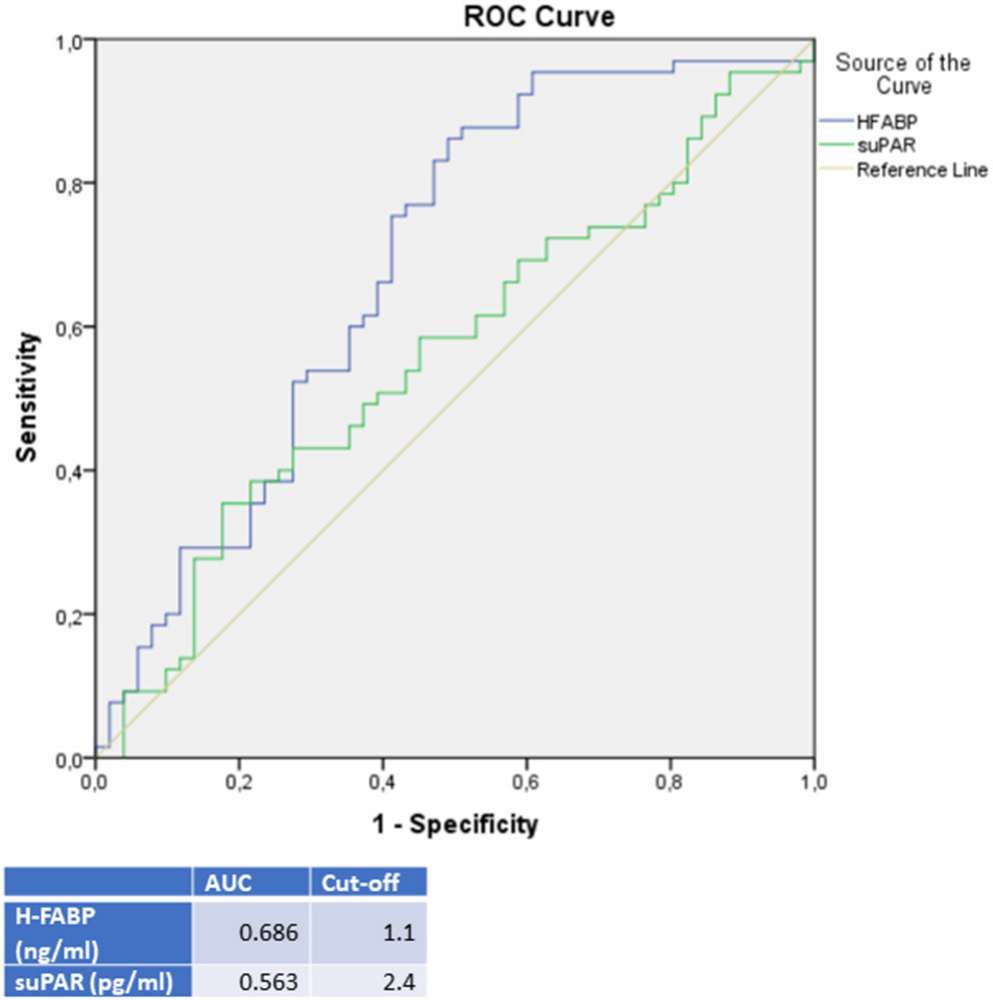
ROC-Curves and cut off scores for H-FABP (HFABP) and suPAR for prediction of DCMP in the total cohort (including patients with DCMP and TTC).

A differential diagnostic value was found for H-FABP in the prediction of a DCMP compared to TTC patients (see Figure 4). A cut off value was given in Figure 4, rates for sensitivity, specifity, positive and negative predictive value were shown in Table 4.

**Table 4:** Rates for sensitivity, specificity, positive and negative predictive value for all tested biomarkers in TTC and DCMP patients.

**TTC**	**Sensitivity**	**Specificity**	**PPV**	**NPV**
sST-2	74.5%	88.9%	82.6%	81.4%
GDF-15	76.5%	47.6%	53.4%	72.1%
DCMP	**Sensitivity**	**Specificity**	**PPV**	**NPV**
H-FABP	87.7%	49.0%	69.1%	74.3%

**Figure 5:**
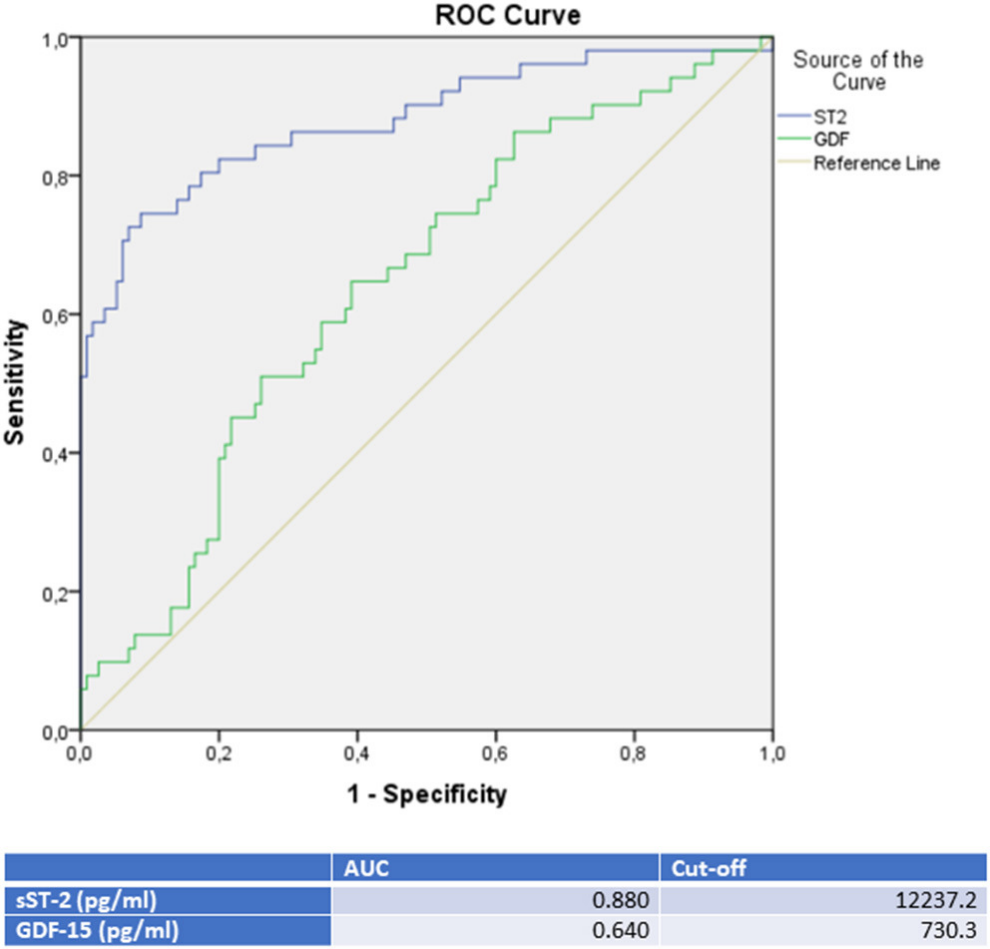
ROC-Curves and cut off scores for sST-2 (ST2) and GDF-15 (GDF) prediction of TTC in the total cohort (including patients with TTC, DCMP, and ICMP).

sST-2 and GDF-15 showed a value to detect TTC patients among a group, including ICMP and DCMP patients (sST-2: *p* = < 0.001, AUC: 0.880; GDF-15: *p* = 0.005, AUC: 0.640). Cut off values were given in Figure 5, rates for sensitivity, specifity, positive and negative predictive value were shown in Table 5.

**Table 5:** Rates for sensitivity, specificity, positive and negative predictive value for all tested biomarkers in TTC and CMP (including ICMP and DMP) patients.

**TTC**	**Sensitivity**	**Specificity**	**PPV**	**NPV**
sST-2	78.4%	84.3%	67.8%	89.9%
GDF-15	64.7%	60.9%	41.8%	79.8%

### Propensity Score Matching

Additionally, we performed propensity score matching for left ventricular ejection fraction, sex and cardiovascular risk factors. Supplementary Figure 1 depicts distribution of propensity scores between the investigated groups before and after propensity score matching, while Supplementary Figure 2 depicts the Love plots after matching.

Notably, in the matched cohort of TTC (*n* = 7) vs. DCMP (*n* = 7), differences in the plasma levels of sST2 and H-FABP remained statistically significant (see Supplementary Table 1). Furthermore, in the matched cohort of TTC (*n* = 7) vs. ICMP (*n* = 7), differences in sST2 remained statistically significant (see Supplementary Table 1).

## Discussion

### Clinical Issue

Among the causes of a *de novo* diagnosed cardiomyopathy, TTC plays a minor relevance with an occurrence of 50,000–100,000 cases per annum in the United states (2, 3). Clinical questions in studies so far, have been focusing on the question of how to differentiate a TTC from an acute coronary syndrome (34). Although in clinical practice, this question remains the predominant clinical issue, TTC patients may solely present with symptoms of *de novo* diagnosed cardiomyopathy too.

After exclusion of a significant coronary artery stenosis, clinical problems focus on a *de novo* diagnosed cardiomyopathy and its management. Some clinical issues raise and have not been answered even by the literature. Large clinical studies on the comparison of TTC with the most frequent types of cardiomyopathies are lacking, despite cardiomyopathies remain the second most important reason for hospitalization (35).

In our study we aimed to analyze the plasma levels of novel cardiovascular biomarkers in most important cardiomyopathies, including ICMP and DCMP, as well as in TTC patients.

Whereas in the acute setting, TTC might be easy identified after the exclusion of a significant coronary artery stenosis, in the subacute setting the evaluation of an apical TTC or atypical types of TTC might be challenging and indicators for a TTC might be of clinical benefit (36). As medical therapy, long term prognosis, rhythm management, recommendations for follow-ups and rehabilitation program differ completely between TTC and ICMP or rather DCMP, biomarkers for differential diagnosis, or rather for confirmation of diagnosis, are warranted.

Further indicators for the genesis of a cardiomyopathy are especially warranted in the differential diagnosis of a TTC compared to an ICMP or a DCMP. Especially when considering that in TTC neurohumoral therapy has proved no endorsed use in clinical studies (19, 37). People might be exposed to adverse events of a long-term neurohumoral treatment despite a described spontaneous remission of 96% in TTC patients within 1 month (36). Even, when a neurohumoral therapy is initiated for empiric short-term therapy in TTC patients, indicators for a discontinuation of neurohumoral therapy after recovery might be warranted to facilitate clinicians' decision.

As life-threatening ventricular arrhythmias occur with a high incidence either in DCMP, ICMP, and TTC, biomarkers as indicators for a classification of unclear cardiomyopathies might be supportive, as rhythm management in TTC differs from ICMP and DCMP. Profound guidelines for the antitachycardia and antibradycardia management in TTC are lacking, but observational studies are available. The indication for implantable cardioverter defibrillator (ICD) implantation for secondary prevention of sudden cardiac death (SCD) might be cautiously seen as spontaneous recovery is reported in 96% of TTC patients within a month. Temporary life securing systems, as successfully reported by wearable cardioverter defibrillator (WCD) in peripartum cardiomyopathy (PPCMP), provide an alternative for SCD prevention unless left ventricular function recovers (38, 39).

Referral to cardiac rehabilitation program is in general low and data of the profit from rehabilitation programs are not secured (40). Therefore, patients can initiate on their own a non-medically surveilled rehabilitation program. Furthermore, in the peripheral hospital without the availability of coronary angiography, coronary CT angiography or cardiac MRI, the triage of cardiomyopathies with unsure origin might be facilitated and immediate transfer to a cardiologic center for further diagnosis might be postponed. Regarding these questions, biomarkers, which allow a better classification of cardiomyopathies with unidentified genesis, are clinical relevance.

### Interpretation of Our Results and Prospective Clinical Implementation

#### sST-2

sST-2 was significantly increased in TTC patients compared to patients with ICMP and DCMP. A ROC analysis of TTC patients compared to ICMP patients (AUC: 0.879; *p* = < 0.001; cut off value: 12,140.5 pg/ml with sensitivity: 78.4%, specifity: 84.6%, PPV: 83.3%, NPV 80.0%), to DCMP patients (AUC: 0.881; *p* = < 0.001; cut off value: 14521.9 pg/ml with sensitivity: 74.5%, specifity: 88.9%, PPV: 82.6%, NPV: 81.4%) or to the combined group of ICMP/DCMP (AUC: 0.880; *p* = < 0.001; cut off value: 12237.2 pg/ml with sensitivity: 78.4%, specifity: 84.3%, PPV: 67.8%, NPV: 89.9%) presented sST-2 as one of the most relevant diagnostic biomarkers in this study for the identification of TTC. In propensity score matching for left ventricular ejection fraction, sex and cardiovascular risk factors, differences in the plasma levels of sST2 in the matched cohort of TTC vs. DCMP and TTC vs. ICMP remained statistically significant. sST-2 had already been investigated to predict the development of stress cardiomyopathy in patients admitted to intensive care units and to stratify in-hospital high risk patients with TTC (41, 42). In our study sST-2 showed no correlation with the left ventricular ejection fraction or plasma creatinine levels. sST-2 in TTC patients may reflect an exposure of mechanical stress and increased neurohormonal activation in these patients. Therefore sST-2 indicates cardiomyocyte strain and hemodynamic stress following apical, midventricular or basal akinesia in the setting of an acute TTC (43).

#### suPAR

Baseline serum plasma levels of suPAR of ICMP patients were at the highest level of the three subgroups, but without a significant difference to TTC patients (*p* = 0.063) and to DCMP patients (*p* = 0.246). These observations are in accordance to our presumptions, as suPAR is reported to be elevated by the formation of atherosclerotic lesions and plaque destabilization (44). In previous reports, high levels of suPAR are described to correlate with the risk of coronary artery disease and matching with our results, suPAR levels were the highest in ICMP patients, followed by DCMP and TTC patients (45).

#### H-FABP

It was of interest, that in our study the highest plasma levels of H-FABP were measured in DCMP patients, followed by the H-FABP levels of ICMP patients and followed with a significant difference to TTC patients (*p* = < 0.001; AUC: 0.686). This observation offers a possible clinical implementation for H-FABP as a marker for differential diagnosis between DCMP and TTC with a cut off value of 1.1 ng/ml (sensitivity: 87.7%, specifity: 49.0%, PPV: 69.1%, NPV: 74.3%). In propensity score matching for left ventricular ejection fraction, sex and cardiovascular risk factors, differences in the plasma levels of H-FABP in the matched cohort of TTC vs. DCMP remained statistically significant. Besides the value of H-FABP as a marker for ischemia and early myocardial damage, H-FABP serves as a parameter for myocardial stress (46). As myocardial stunning is the driving pathogenesis in TTC, less myocardial stress seems to be present in TTC patients compared to clinically compensated DCMP patients (47).

#### GDF-15

The highest GDF-15 levels were measured in TTC compared to ICMP (AUC: 0.626, *p* = 0.028), to DCMP (AUC: 0.653, p = 0.007) and to the combined group of ICMP/DCMP (*p* = 0.005; AUC: 0.640), indicating a differential diagnostic value. The cut off value of GDF-15 for the identification of TTC compared to ICMP was 537.7 pg/ml (sensitivity: 86.4%, specifity: 36.5), 608.2 pg/ml (sensitivity: 76.5%, specifity: 47.6%) for the prediction of a DCMP and 730.3 pg/ml (sensitivity: 64.7%, specifity: 60.9%) for a differentiation to the combined group of ICMP/DCMP. Higher GDF-15 levels had already been analyzed in a study of 22 TTC patients compared to ACS patients (48). GDF-15 had been described as a stress-responsive biomarker of cardiac and vascular disease. GDF-15 expression was up-regulated in the presence of oxidative stress and inflammation, which is in accordance to previous reports indicating that inflammation and oxidative stress are driving pathogenicity factors of TTC (49, 50).

## Conclusion

Novel cardiovascular biomarkers such as GDF-15, H-FABP and sST-2 offer a differential diagnostic value for distinguishing between TTC, DCMP or ICMP and could help in the identification of unclear cardiomyopathies. Therefore, the guidance of treatment might be facilitated, as medical therapy, long term prognosis, rhythm management, recommendations for follow-up and rehabilitation program differ completely between TTC and ICMP or rather DCMP.

## Limitations

Major limitations of the present study are the relatively small study cohort. Hence, the findings of our study have to be confirmed in large-scale studies to confirm the results of the present study.

## Data Availability Statement

The raw data supporting the conclusions of this article will be made available by the authors, without undue reservation.

## Ethics Statement

The studies involving human participants were reviewed and approved by Ethics Committees of the University Salzburg and Jena. The patients/participants provided their written informed consent to participate in this study.

## Author Contributions

AT, RP, and ML designed the study. AT, MM, NB, and BO wrote the manuscript. VP and JG performed laboratory analyses. RL and ML provided assistance and revised the manuscript. UH provided resources. All authors contributed to the article and approved the submitted version.

## Funding

Funding for the study was provided by Department of Internal Medicine II, Paracelsus Medical University, Austria.

## Conflict of Interest

The authors declare that the research was conducted in the absence of any commercial or financial relationships that could be construed as a potential conflict of interest.

## Publisher's Note

All claims expressed in this article are solely those of the authors and do not necessarily represent those of their affiliated organizations, or those of the publisher, the editors and the reviewers. Any product that may be evaluated in this article, or claim that may be made by its manufacturer, is not guaranteed or endorsed by the publisher.
