# Supplementary material for: Analysis of Selected Cardiovascular Biomarkers in Takotsubo Cardiomyopathy and the Most Frequent Cardiomyopathies
Source: Front Cardiovasc Med. 2021 Nov 3;8:700169. doi: 10.3389/fcvm.2021.700169 (PMC8597641; doi:10.3389/fcvm.2021.700169)
Supplement: Supplementary file 1 [file Data_Sheet_1.docx]

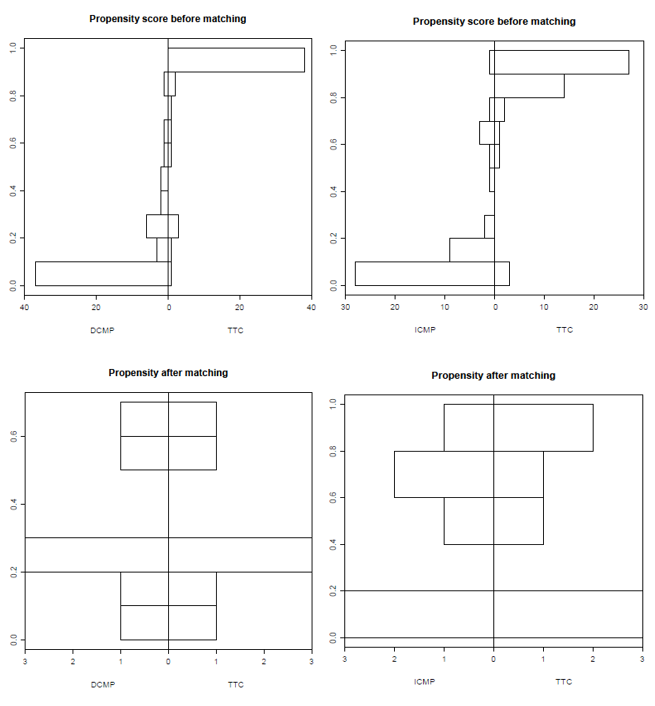


**Suppl. Figure 1.** Propensity score distribution before/after matching.


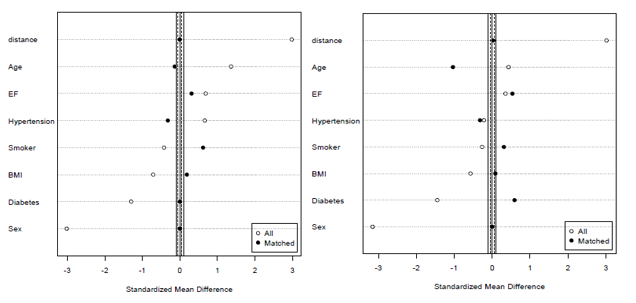


**Suppl. Figure 2.** Love Plot TTC/DCMP (left) and Love Plot TTC/ICMP (right) after matching.

|  | TTC |  | DCMP |  | *P*= | TTC |  | ICMP |  | *P=* |
| --- | --- | --- | --- | --- | --- | --- | --- | --- | --- | --- |
|  | Median | IQR | Median | IQR |  | Median | IQR | Median | IQR |  |
| EF (%) | 45.0 | 25.0 -50.0 | 45.0 | 20.0 -47.0 | 0.653 | 45.0 | 25.0 -50.0 | 30.0 | 25.0 -51.0 | 0.607 |
| Age (y) | 59.0 | 51.0-64.0 | 55.0 | 51.0-69.0 | 0.847 | 60.0 | 53.0-64.0 | 72.0 | 66.0-79.0 | 0.035 |
| Sex (female,n) | 4/7 (57.1%) |  | 4/7  (57.1%) |  | 0.990 | 4/7  (57.1%) |  | 4/7  (57.1%) |  | 0.990 |
| BMI (kg/m^2) | 28.4 | 21.8-34.0 | 27.7 | 24.1-29.4 | 0.565 | 28.4 | 21.8-30.4 | 27.3 | 24.1-31.6 | 0.949 |
| Smoker (n) | 3/7 (42.9%) |  | 1/7 (14.3%) |  | 0.514 | 2/7 (28.6%) |  | 1/7 (14.3%) |  | 0.794 |
| Hypertension (n) | 3/7 (42.9%) |  | 4/7 (57.1%) |  | 0.068 | 5/7 (71.4%) |  | 6/7 (85.7%) |  | 0.501 |
| Diabetes (n) | 3/7 (42.9%) |  | 3/7 (42.9%) |  | 0.990 | 3/7 (42.9%) |  | 2/7 (28.6%) |  | 0.043 |
| sST-2 (pg/ml) | 23592.2 | 17796.5 -31402.3 | 10382.7 | 6484.1 -11346.6 | 0.003 | 17796.5 | 15100.6 -31402.3 | 6031.7 | 5529.7 -9180.5 | 0.002 |
| H-FABP (ng/ml) | 1.6 | 0.4-1.0 | 3.1 | 1.5-3.7 | 0.006 | 0.7 | 0.5-1.3 | 1.7 | 0.0-2.0 | 0.949 |
| suPAR (pg/ml) | 3118.8 | 2311.2-3706.2 | 3014.4 | 2349.4-4897.3 | 0.749 | 3441.9 | 2760.1-3909.8 | 3630.8 | 2216.0-7604.1 | 0.848 |
| GDF-15 (pg/ml) | 931.0 | 420.3-2204.8 | 517.6 | 448.0-2132.7 | 0.848 | 592.9 | 493.7-1565.3 | 624.4 | 297.4-1426.2 | 0.565 |

**Suppl. Table 1.** Propensity score matching for left ventricular ejection fraction, sex and cardiovascular risk factors.
